# Discovery and validation of a colorectal cancer classifier in a new blood test with improved performance for high-risk subjects

**DOI:** 10.1186/s12014-017-9163-z

**Published:** 2017-07-25

**Authors:** Lisa J. Croner, Roslyn Dillon, Athit Kao, Stefanie N. Kairs, Ryan Benz, Ib J. Christensen, Hans J. Nielsen, John E. Blume, Bruce Wilcox

**Affiliations:** 1 Applied Proteomics, Inc, 3545 John Hopkins Court, Suite 150, San Diego, CA 92121 USA; 2 Department of Surgical Gastroenterology 360, Hvidovre Hospital, University of Copenhagen, 2650 Hvidovre, Denmark

**Keywords:** Blood tests, Clinical markers, Colorectal neoplasms, Tumor biomarkers, Proteomics

## Abstract

**Background:**

The aim was to improve upon an existing blood-based colorectal cancer (CRC) test directed to high-risk symptomatic patients, by developing a new CRC classifier to be used with a new test embodiment. The new test uses a robust assay format—electrochemiluminescence immunoassays—to quantify protein concentrations. The aim was achieved by building and validating a CRC classifier using concentration measures from a large sample set representing a true intent-to-test (ITT) symptomatic population.

**Methods:**

4435 patient samples were drawn from the *Endoscopy II* sample set. Samples were collected at seven hospitals across Denmark between 2010 and 2012 from subjects with symptoms of colorectal neoplasia. Colonoscopies revealed the presence or absence of CRC. 27 blood plasma proteins were selected as candidate biomarkers based on previous studies. Multiplexed electrochemiluminescence assays were used to measure the concentrations of these 27 proteins in all 4435 samples. 3066 patients were randomly assigned to the Discovery set, in which machine learning was used to build candidate classifiers. Some classifiers were refined by allowing up to a 25% indeterminate score range. The classifier with the best Discovery set performance was successfully validated in the separate Validation set, consisting of 1336 samples.

**Results:**

The final classifier was a logistic regression using ten predictors: eight proteins (A1AG, CEA, CO9, DPPIV, MIF, PKM2, SAA, TFRC), age, and gender. In validation, the indeterminate rate of the new panel was 23.2%, sensitivity/specificity was 0.80/0.83, PPV was 36.5%, and NPV was 97.1%.

**Conclusions:**

The validated classifier serves as the basis of a new blood-based CRC test for symptomatic patients. The improved performance, resulting from robust concentration measures across a large sample set mirroring the ITT population, renders the new test the best available for this population. Results from a test using this classifier can help assess symptomatic patients’ CRC risk, increase their colonoscopy compliance, and manage next steps in their care.

## Background

Colorectal cancer (CRC) is a broadly occurring and lethal cancer, with approximately 1.4 million new cases and 700,000 deaths yearly [1]. CRC outcome dramatically improves with early detection followed by curative resection [2–4]; thus CRC screening is recommended for all U.S. patients over 50 years of age [5, 6]. The gold standard screening test is colonoscopy, with some stool-based tests also having good performance [7, 8]. However, compliance with CRC screening recommendations is low; by some measures only 40% of the population for which screening is recommended will undergo testing [9].

A low-burden CRC screening test, such as a blood-based test, has been widely sought. However, it has proven difficult to find blood-based CRC signal with performance matching that of colonoscopy or of stool-based tests across average risk patients. Blood-based CRC signal may be stronger in patients with more advanced disease, such as those with symptoms of colorectal neoplasia. If so, the appearance of symptoms would offer an opportunity to provide low-burden CRC testing with higher performance. Interest in a low-burden CRC test for symptomatic patients has also come from the clinical community. By itself, the increased CRC prevalence in the symptomatic population (10.9% in a Danish symptomatic cohort [10], as compared to 0.5–0.7% in the average risk population [7, 9]) would seem sufficient incentive for patients to follow clinicians’ recommendations to have colonoscopies. However, the compliance rate in the symptomatic population is estimated to be only 63.6% (unpublished observations). A low-burden CRC test for this population would highlight patient risk stratification, resulting in increased personalized incentive and increased colonoscopy compliance [11, 12].

Given the attractiveness of a low-burden CRC test for symptomatic patients, several groups have focused efforts here [10, 11, 13, 14]. The highest performing test, and the only validated test to date, was a blood-based test developed earlier in our laboratory [10, 14] using a sample set mirroring the composition of the intent-to-test (ITT) symptomatic population [14]. The specific symptoms present in this population (abnormal bowel habits, abdominal pain, rectal bleeding, unexplained weight loss, meteorism, anemia, and/or palpable mass) indicated a likelihood of increased CRC risk, which was borne out by the study colonoscopy results; hence we term these patients “high risk.” The positive predictive value (PPV) of this test was 31%, meaning that 31% of the patients with positive test results had CRC uncovered during colonoscopies. This was a dramatic improvement over the positive CRC rate from asymptomatic screening colonoscopy alone (0.5–0.7%, [7, 9]) and over the positive CRC rate within the symptomatic population without additional stratification (10.9%, [10]). The strong performance of this earlier test demonstrated that a low-burden test for symptomatic patients provides information that may dramatically improve colonoscopy compliance among these high risk patients—a positive result would indicate much more certainty about the usefulness of further testing.

In the present paper, we report the development of a new blood-based CRC test. The new test is directed to the ITT population of symptomatic patients, and was developed using a much larger sample set (4435 vs 922 patient samples) and employing an assay format with more robust analytic performance: electrochemiluminescence antibody-based assays. These assays offer greater dynamic ranges and higher sensitivities when compared to the ELISA format used in the earlier test [15]. The new test’s validated classifier algorithm, developed using feature selection and machine learning applied to concentration measures of 27 proteins in the 4435 patient sample set, has significantly better specificity, resulting in a higher PPV. The new test offers a low-burden, high quality, and high performance CRC risk assessment to clinicians serving patients presenting with CRC symptoms. Results can be used to manage these patients’ choices about further CRC testing.

## Methods

### Patient samples

Patient samples were drawn from a high quality clinical sample set, *Endoscopy II*, described previously [10]. Briefly, samples were collected at seven hospitals across Denmark between 2010 and 2012. Samples were obtained from 4698 subjects presenting with symptoms of colorectal neoplasia (abnormal bowel habits, abdominal pain, rectal bleeding, unexplained weight loss, meteorism, anemia, and/or palpable mass) and scheduled for first-time colonoscopies. The colonoscopies revealed the presence or absence of CRC and/or polyps, with CRC staged according to the UICC TNM system [16].

Each *Endoscopy II* patient was placed in one of eight diagnostic groups based on colonoscopy results and comorbidities: colon cancer (all stages), rectal cancer (all stages), colon adenoma, rectal adenoma, no comorbidities and no CRC or polyps (“no comorbidity-no finding” group), comorbidities present and no CRC or polyps (“comorbidity-no finding” group), other cancer(s), or other colonoscopy findings (“other findings”) [10]. “Comorbidity” refers to Crohn’s disease, colitis, diverticulitis, acute chronic inflammation, diabetes, rheumatoid arthritis, cardiovascular diseases, cirrhotic liver diseases, obstructive lung diseases, and/or restrictive lung diseases. The entire sample set represented the composition of a target population of patients at high risk of CRC because of their symptomology.

A total of 4435 *Endoscopy II* samples was used in the current study. These were selected from the total of 4698 samples based on available sample volume. The proportions of diagnostic groups in the study reflected those of the high-risk population represented by the entire sample set. Table 1 presents the study samples’ characteristics under the column entitled “Endo II.”

**Table 1:** Study subject characteristics for *Endoscopy II* overall, and for the current study subsets

	Endo II	CRC ITT discovery set		CRC ITT validation set	
	All	Control	Disease	Control	Disease
Total	4698	2759	340	1189	147
*Clinic number: #*					
31	605	340	53	135	26
32	299	171	13	81	9
33	966	579	71	249	21
34	300	190	18	65	12
35	957	564	72	240	34
36	858	510	48	248	17
37	713	405	65	171	28
*Age: years*					
Mean	63.5	62.7	69.7	62.9	70.1
Standard deviation	12.6	12.6	10.6	12.7	10.7
Median	64.3	63.6	69.5	63.3	71.5
Minimum	18.1	20.1	37.5	18.1	23.6
Maximum	96.0	96.0	94.8	93.3	89.1
*Gender: # (%)*					
Female	2455 (52.3)	1473 (53.4)	144 (42.4)	650 (54.7)	55 (37.4)
Male	2243 (47.7)	1286 (46.6)	196 (57.6)	539 (45.3)	92 (62.6)
*BMI: kg/m* ^*2*^					
Mean	25.6	25.6	25.5	25.5	26.2
Standard deviation	4.6	4.7	4.7	4.5	3.9
Median	25.1	25.1	24.8	25.0	25.7
Minimum	11.7	13.0	15.8	11.7	16.9
Maximum	50.5	50.2	47.1	50.5	39.1
*CRC stage: # (%)*					
I	101 (19.8)	0 (0)	74 (21.8)	0 (0)	25 (17.0)
II	163 (31.9)	0 (0)	105 (31.0)	0 (0)	50 (34.0)
III	139 (27.2)	0 (0)	87 (25.7)	0 (0)	45 (30.6)
IV	108 (21.1)	0 (0)	73 (21.5)	0 (0)	27 (18.4)
*Diagnosis: # (%)*					
Colon cancer	319 (6.8)	0 (0)	211 (62.1)	0 (0)	92 (62.6)
Rectal cancer	193 (4.1)	0 (0)	129 (37.9)	0 (0)	55 (37.4)
Adenoma colon	515 (11.0)	340 (12.3)	0 (0)	148 (12.4)	0 (0)
Adenoma rectum	174 (3.7)	117 (4.2)	0 (0)	51 (4.3)	0 (0)
No comorbidity-no finding	1164 (24.8)	763 (27.7)	0 (0)	334 (28.1)	0 (0)
Comorbidity-no finding	814 (17.3)	534 (19.4)	0 (0)	229 (19.3)	0 (0)
Other cancer	177 (3.8)	119 (4.3)	0 (0)	50 (4.2)	0 (0)
Other finding	1342 (28.6)	886 (32.1)	0 (0)	377 (31.7)	0 (0)

### Candidate biomarkers

Twenty-seven blood plasma proteins were selected as candidate biomarkers for this study. These markers were selected based on (1) their performance in a previous study wherein 187 CRC-related proteins were assessed for CRC signal in a high-multiplex, targeted-mass spectrometry discovery and validation study [17], (2) additional literature review identifying TFF3 and TFRC as new candidates [18, 19], (3) commercial availability of high quality antibodies for those proteins identified as promising CRC biomarkers, and 4) the successful development of multiplexed electrochemiluminescence assays. Table 2 lists the 27 proteins measured across five multiplexed panels. The commercially available antibody pairs selected to measure each of the 27 proteins did not distinguish between different isoforms of the proteins.

**Table 2:** Protein targets across the five multiplexed panels, showing allowable ranges of analytical parameters

Panel	Protein	Abbreviation	Dilution factor	Duplicate CV max (%)	Hill slope min	Hill slope max	LLoQ (pg/mL)	ULoQ (pg/mL)
1	Alpha-1-acid glycoprotein	A1AG	300,000	20	0.9	1.1	12.21	50,000
1	Alpha-1 antitrypsin	A1AT	300,000	20	0.9	1.1	73.24	300,000
1	Apolipoprotein A-I	APOA1	300,000	20	0.9	1.1	244.14	1,000,000
1	Complement 3	CO3	300,000	20	0.9	1.1	610.35	2,500,000
1	Haptoglobin	HPT	300,000	20	0.9	1.1	488.28	2,000,000
2	Alpha-antichymotrypsin	AACT	5000	20	0.6	0.8	1220.7	5,000,000
2	Carbonic anhydrase 1	CAH1	5000	20	0.9	1.1	2.44	10,000
2	Clusterin	CLUS	5000	20	0.8	1.2	244.14	1,000,000
2	Complement 9	CO9	5000	20	0.9	1.2	24.41	100,000
2	C-reactive protein	CRP	5000	20	0.9	1.1	12.21	50,000
2	Dipeptidyl peptidase IV	DPPIV	5000	20	0.9	1.1	2.44	10,000
2	Serum amyloid A	SAA	5000	20	0.9	1.1	12.21	50,000
2	Transferrin receptor protein	TFRC	5000	20	0.9	1.1	0.49	2000
3	Protein S100-A8/-A9	CALP	100	20	1.1	1.6	48.83	200,000
3	Cathepsin D	CATD	100	20	0.9	1.1	19.53	80,000
3	Growth differentiation factor 15	GDF15	100	20	0.9	1.1	0.12	500
3	Gelsolin	GELS	100	20	0.85	1.1	488.28	2,000,000
3	Prolyl endopeptidase FAP	SEPR	100	20	0.85	1.15	2.44	10,000
3	Tissue metalloproteinase inhibitor 1	TIMP1	100	20	1.3	1.5	12.21	50,000
4	Annexin A1	ANXA1	4	20	0.9	1.1	12.21	50,000
4	Carcinoembryonic antigen-related cell adhesion molecule 5	CEA	4	20	0.9	1.1	24.41	100,000
4	Glycine-tRNA ligase	GARS	4	20	0.9	1.1	122.07	500,000
4	Macrophage migration inhibitory factor	MIF	4	20	0.9	1.1	14.65	60,000
4	Trefoil factor 3	TFF3	4	20	0.9	1.1	0.49	2000
5	Pyruvate kinase isozyme M2	PKM2	4	20	0.9	1.2	7812.5	2,000,000
5	Peroxiredoxin-1	PRDX1	4	20	0.9	1.1	12.21	50,000
5	P-selectin glycoprotein ligand 1	PSGL	4	20	0.9	1.1	12.21	50,000

Observed lower and upper limits of quantitation (LLoQ and ULoQ) are listed as concentrations of diluted samples

### Assays

Multiplexed electrochemiluminescence immunoassays (Meso Scale Discovery, Rockville, MD) were used to assess the concentrations of each of the 27 proteins in all 4435 samples of the study.

#### Custom assay development

A feasibility study was conducted to develop multiplexed immunoassays on the electrochemiluminescence platform for proteins uncovered in previous CRC biomarker studies [10, 17] and candidate CRC-related biomarker proteins identified from literature searches [18, 19]. During the feasibility study, extensive screening of commercially available antibodies and standards was performed. The antibodies, standards, and diluents for each assay were selected based on pre-established analytical performance criteria related to the affinity and specificity for the target protein. These criteria were assessed by experiments exploring linearity of sample matrix dilution, calibrator spike and recovery, and antibody-target dissociation rates. Assays that failed to meet the criteria were deemed unreliable and were excluded from further development. Of the proteins identified in previous biomarker studies [10, 17], alpha-Amylase 2b (AMY2B), Delta(3,5)-delta(2,4)-dienoyl-CoA isomerase mitochondrial (ECH1), Ferritin light chain (FRIL), Osteopontin (OSTP), Selenium-binding protein 1 (SBP1), and Spondin-2 (SPON2) were excluded from final assay development on the electrochemiluminescence platform due to either failure to meet the feasibility performance criteria or the lack of high quality commercially available antibodies.

Following identification of a reliable antibody source and demonstration of analytical performance, assays were further developed and optimized for the 27 candidate biomarkers (Table 2). During development, the dynamic range and linearity of each assay were established using the intended biological matrix, human plasma. The assay workflow for five multiplexed panels (with 3–8 analytes per panel) encompassing the 27 analytes was transferred to automated liquid handling systems. Automation of the multiplexed assay workflow facilitated high-throughput sample processing and ensured maximal accuracy and precision over the course of the study.

#### Five multiplexed assay panels

Each assay panel was run in 96-well plates. Standard curves (seven standards and a blank), process quality controls (PQCs, from pooled human plasma samples, BioreclamationIVT, Westbury, NY), and patient samples were tested in duplicate on each plate. A single run required 110 μl of plasma to test all 27 analytes in five multiplexed panels. A single lot of all assay materials (antibodies, standards, plates and diluents) was used to minimize variation across the study. Standard preparations and sample dilutions were performed on a Tecan Freedom EVO (Tecan, Männedorf, Switzerland). Following plating of the standards and samples on the Tecan Freedom EVO, the reagent additions and wash steps were performed on a BioTek EL406 Washer Dispenser (BioTek, Winooski, VT). The levels of electrochemiluminescent units, corresponding to analyte concentration, were measured on a QuickPlex SQ 120 Imager (Meso Scale Discovery, Gaithersburg, MD) using MSD Discovery Workbench software with 4 parameter logistic curve fitting and 1/y^2^ weighting. The test assays are listed in Table 2, which also gives the panel compositions, sample dilutions, maximum allowable duplicate CVs, minimum and maximum allowable Hill slopes, and observed lower and upper limits of quantitation (LLoQ and ULoQ). Acceptance criteria were established in accordance with FDA guidance on bioanalytical method validation [20]. The acceptance criteria for assay plates included standard curve quality (Hill slope within limits, R^2^ ≥ 0.95), PQC analyte concentrations (within pre-established ranges), and PQC duplicate CVs (below CV maxima). Sample measures from plates passing acceptance criteria were accepted if their duplicate CVs were below the CV maxima.

The decision to use the electrochemiluminescence platform was driven by the advantages of this format. Customized development of assays for the protein targets enabled selection of new antibodies, standards, and diluents that provided maximum specificity and selectivity in a multiplexed format. The electrochemiluminescence gave excellent sensitivity for each assay, typically a 3–4 log dynamic range. Multiplexing allowed for simultaneous measurement of 3–8 analytes from a single reaction volume. Also, the electrochemiluminescence format typically required 50% of the plasma volume and shorter assay times relative to other immunoassay platforms.

### Classifier construction and statistical analysis

#### Study design

The study goals were to uncover a panel of biomarkers (including plasma protein concentrations and possibly demographic features) and a CRC classifier model, such that the biomarkers’ values would serve as predictors in the classifier algorithm to distinguish CRC (all stages) from non-CRC patients in the high-risk ITT population. This goal was approached using a standard machine learning study design: biomarker panels and classifier models were developed in a Discovery set. The combined panel and algorithm with the most promising performance at differentiating CRC from non-CRC was then tested in a separate Validation set.

Both the Discovery and Validation sets were built to represent the ITT population of symptomatic patients in the *Endoscopy II* study. For both sets, samples were selected at random across the eight diagnostic groups so that the proportions of different diagnostic groups matched those in the entire *Endoscopy II* sample set (see Table 1, columns entitled “CRC ITT Discovery Set” and “CRC ITT Validation Set”). To further ensure that the Discovery and Validation sets represented the ITT population, no attempt was made to artificially balance patient characteristics across disease and control classes. Thus patient characteristics such as age and gender, which are known to be correlated with CRC [e.g. 5, 6], were allowed to vary naturally between the classes.

The Discovery set consisted of 3099 samples, while the Validation set consisted of 1336 samples. The Discovery and Validation sets were completely independent, with no overlap of samples between the two sets.

#### Classifier discovery and validation

Biomarker panels and classifier algorithms were explored extensively in the Discovery set. Classifiers were built to distinguish CRC patients (all stages pooled) from non-CRC patients, with no filtering of the non-CRC groups; thus they were built to distinguish CRC of any stage in the true ITT population. The performance target was sensitivity/specificity of at least 0.80/0.80.

We employed machine learning approaches covering a diverse set of methods for both predictor selection and classifier modeling. In machine learning studies for which domain knowledge reveals a clear and well-established mechanism driving relationships between predictors and outcome classes, the selection of machine learning methods can be straightforward. For example, linear feature selection and classifier models will perform well when a weighted combination of predictors has a straightforward relation to the outcome classes. However, if several different linear relations have been observed conditional on the status of a subset of predictors, or if more complex if–then processes best describe the relations between predictors and classes, then decision trees are likely a better option. On the other hand, when domain knowledge is either lacking or reveals that diverse and complex mechanisms drive relationships between predictors and outcome classes, it is best to explore a wide range of machine learning approaches. This is the case with the biology of cancer. In this study, we therefore utilized a wide range of machine learning methods.

To achieve this, a grid search was used to examine many combinations of data type, data pre-treatment, predictor number, feature selection algorithm, and classifier algorithm. Data types included protein concentrations as well as protein concentration ratios; age and gender were also included, with gender represented as a binary numerical variable. Data pre-treatment options included log2-transformation of concentrations, and/or concentration standardization (zero mean, unit variance). All possible concentration ratios were added as individual predictors to some classifier builds, with the ratios undergoing the same data pre-treatments. Classifiers were built using 2–29 predictors. Feature selection algorithms included Elastic Net, Linear Correlation, Rank Correlation, Information Gain, Gain Ratio, Random Forest Accuracy, and Random Forest Impurity. Classifier algorithms included Logistic Regression, Elastic Network Regression, Support Vector Machines, Boosting, Random Forests, and K Nearest Neighbor models; in addition, a variety of parameters was investigated for each algorithm. For each combination of data type and feature selection algorithm, the classifier grid explored every possible combination of data pre-treatments, predictor numbers, classifier algorithms, and classifier algorithm parameters. For each combination, a strict ten-fold cross-validation procedure was repeated ten times. Performance was calculated for each replicate as the performance seen in the combined results from the ten folds’ hold-out test sets, and then summarized as the median across replicates. Classifier performances were compared across all builds to select those with the highest cross-validation AUCs in the Discovery set. These classifier candidates were then filtered based on predictor count to select the single model with the fewest protein predictors; this was the top candidate model.

Next the model was refined to improve performance in the target ITT population represented by the full Discovery set. Specifically, Indeterminate score ranges enabling 15, 20, or 25% Indeterminate rates were explored. (Some clinical diagnostic tests employ an Indeterminate score range [e.g. 21]. Patients with scores in this range would not receive a model call.) Optimal Indeterminate score ranges were found by applying the model to measures from all Discovery set samples, then examining all possible Indeterminate score ranges that removed 15, 20, or 25% of the samples. For each range specification, the Indeterminate score range that gave the maximum specificity with sensitivity above 0.80 was selected. Indeterminate specifications and ranges that enabled the target performance were then selected. Considerations of combined performance and acceptable Indeterminate specifications led to selection of one particular range. The classifier model and Indeterminate range were locked at this point, marking the end of the classifier discovery process.

The locked model, along with the Indeterminate range, was then applied directly to the separate Validation set. Validation was considered successful if the classifier performance in the Validation set was (1) statistically indistinguishable from that observed in the Discovery set and/or (2) above the performance sensitivity/specificity target of 0.80/0.80.

#### Software and statistical tests

All analyses were performed using the R programming language running in Unix and OSX environments [22]. The grid search code was developed in-house, and run parallelized across multiple compute servers. Most feature selection algorithms were drawn from the FSelector package [23]; some were constructed using the randomForest [24] or glmnet [25] packages. Classifier algorithms were drawn from the randomForest [24], glmnet [25], e1071 [26], kknn [27], and mboost [28] packages. The ROCR and pROC packages were used to calculate model performance and to statistically compare performances [29, 30]. DeLong’s test was used to compare AUCs from ROCs [31]. Fisher’s exact test was used to analyze contingency tables [32].

## Results

Our classifier discovery procedure yielded nine CRC versus non-CRC classifiers with median cross-validation AUCs of 0.84 or higher. Four of these classifiers were dropped from consideration because they included one or both of two assays for which continued availability of reagents was uncertain (GARS and CALP). Of the remaining five classifiers, the one with the fewest protein predictors was selected as the top candidate. This model’s algorithm was a logistic regression using eight protein concentrations (log2 transformed, unscaled, selected using penalized regression [GLMNet]-based ranking), age, and gender as predictors of CRC status. The eight proteins were A1AG, CEA, CO9, DPPIV, MIF, PKM2, SAA, and TFRC. An Indeterminate score range removing 25% of the Discovery set samples was selected on the basis of performance and market acceptability. In the Discovery set, application of this Indeterminate range yielded an AUC of 0.89 and a sensitivity/specificity point of 0.80/0.87. The model, along with the Indeterminate score range, was locked at this point.

**Fig. 1:**
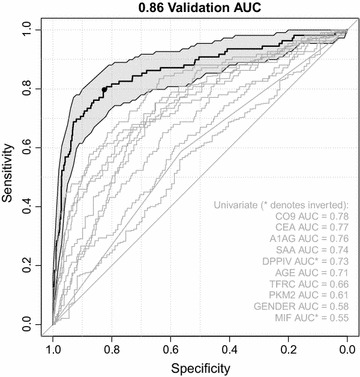
Validation ROC (*black curve*). The Validation AUC was 0.86 with a 95% confidence interval of 0.82–0.90 (*shaded region*). The selected sensitivity/specificity point was 0.80/0.83 (*black dot*). ROCs for each of the single model predictors are also shown (*gray curves*, univariate AUCs listed in the figure)

The locked model was then applied to the Validation set. There were 310 Validation set samples with scores in the Indeterminate range (23.2%, 95% confidence interval 21.0–25.6%). There was no significant difference between the counts of CRC and non-CRC patients in these Indeterminate samples as compared to the full ITT population (Fisher’s test, *p* = 0.549). Figure 1 shows the Validation set ROC. The Validation set ROC was not statistically distinguishable from the Discovery set ROC (DeLong’s test on the ROC AUCs, *p* = 0.503). The Validation AUC was 0.86 (95% confidence interval 0.82–0.90). A performance point of sensitivity/specificity 0.80/0.83 was selected as that meeting our target performance of 0.80/0.80 or better. Figure 1 also shows the Validation set ROCs and AUCs for each of the individual predictors alone (outside of the classification model). The clear segregation of the individual predictors’ ROCs from the classifier model ROC demonstrates that (1) the model is not overly dependent on any one predictor, and (2) it is the model’s algorithmic combination of predictors into a single score that gives it improved discriminatory power over single markers.

Table 3 is a confusion matrix showing results from the Validation set. Table 4 offers a summary of the model’s final performance parameters. With a 10.9% CRC prevalence in the symptomatic population [10], the positive predictive value (PPV) was 36.5% and the negative predictive value (NPV) was 97.1%.

**Table 3:** Confusion matrix from the validation set

	Model call		Total
	Non-CRC	CRC	
*Patient status*			
Non-CRC	758	159	917
CRC	22	87	109
Total	780	246	1026

**Table 4:** The final model’s performance parameters

Sensitivity	0.80
Specificity	0.83
Positive predictive value (PPV)	36.5%
Negative predictive value (NPV)	97.1%

### Early and late stage CRC

Ideally, a CRC test would detect early stage CRC so that interventions can be offered before the cancer progresses. The final model presented here was built to distinguish CRC of any stage from non-CRC—no special models were built to distinguish either early or late stage CRC separately. However, since CRC signal may be stronger in more advanced stages, the model could have been driven primarily by signal in the patients with later stage CRC lesions. If that were the case, the model’s sensitivity to early and late stage CRCs would differ. To explore this possibility, the final model’s sensitivity to early (stages I–II) and late (stages III–IV) stage CRC is shown in Table 5. Sensitivities for early and late stages were not significantly different (binomial test *p* = 0.1115), and there was no significant association between cancer stage and call correctness (Fisher’s test, *p* = 0.338); thus there was no evidence of different classification performance for early and late stage CRC.

**Table 5:** The final model’s CRC sensitivities for early and late stage cancer

CRC stage	Incorrect call	Correct call	Sensitivity
I–II	12	36	0.75
III–IV	10	51	0.84
Total	22	87	0.80
Fisher’s test *p* value	0.338		

## Discussion

### Comparison to an earlier mass spectrometry CRC panel from our laboratory

Earlier work from our laboratory used targeted-mass spectrometry to explore CRC signal in plasma samples [17]. That study uncovered a set of candidate CRC biomarkers of which 15, from 13 proteins, were combined in a classifier to predict CRC in asymptomatic patients. In translating these early mass spectrometry discoveries into a viable clinical test, commercial considerations (see below) dictated the choice of immunoassays using commercially available antibodies. Because of the variable affinity and specificity of commercial antibodies, there was no assurance that the exact 13 protein panel outlined in the targeted-mass spectrometry study would translate successfully to immunoassays. In addition, the ITT group of symptomatic patients differed from the asymptomatic patients used in the mass spectrometry study. Therefore, to ensure that the final test would work well using immunoassays in the ITT population, additional development and studies were undertaken.

In developing the electrochemiluminescence assays of the current study, high quality antibodies and calibrators were identified for seven of the 13 proteins from the targeted-mass spectrometry study—A1AG, A1AT, CLUS, CO9, GELS, SEPR, and TIMP1. For the remaining six proteins—AMY2B, ECH1, FRIL, OSTP, SBP1, and SPON2—successful electrochemiluminescence immunoassays were not developed. Since the 13 proteins had been a subset of those carrying CRC signal in the mass spectrometry study, it was reasonable to expect that a new immunoassay-based panel with good performance could be found by drawing from a broader set of the biomarker candidates uncovered in the mass spectrometry study. Therefore, we set out to assess the 27 biomarker candidates of the current study—25 that translated successfully from mass spectrometry to electrochemiluminescence assays, and two new proteins—aiming to uncover a novel panel and develop a new classifier that functioned well in the ITT population.

Hence, because of both the change in assay format and the new patient population, the panel composition and classifier algorithm are different from those presented in the earlier mass spectrometry study.

### Comparison to an earlier immunoassay CRC panel from our laboratory

After our original targeted-mass spectrometry study in CRC patients [17], our first immunoassay test for the CRC-symptomatic population was developed using ELISA assays [14]. Specifically, we uncovered an eight protein ELISA panel that, when combined with age, discriminated CRC from non-CRC with lower specificity and lower PPV than the new biomarker panel presented here. The ELISA CRC panel differed from the new panel presented here in both protein composition and assay technology (ELISA vs electrochemiluminescense assays).

**Fig. 2:**
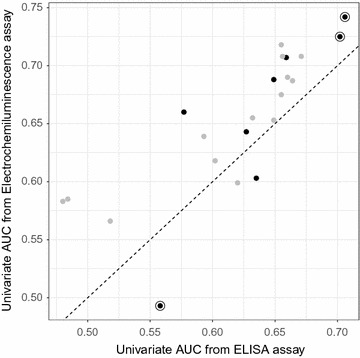
Scatterplot of univariate AUCs for discriminating CRC from non-CRC in the full Discovery set of the current study versus the ELISA study [14]. *Black dots* show the eight proteins used in the current study’s final model; *circles* identify the proteins used in both the current and the ELISA panel. The identity line is *dashed*

In order to understand what drives the new panel’s improved performance, it’s useful to consider the CRC signal carried by each of the individual proteins measured during development of both immunoassay panels. These data are presented in Table 6 for the three proteins that appear in both panels, and in Fig. 2 for the 22 proteins that were assessed in both studies. Figure 2 demonstrates that most of the univariate AUCs obtained with the electrochemiluminescence assays exceeded those obtained with the ELISAs. Six of the proteins used in the new panel (black dots) were among this majority, while two had lower univariate AUCs than measured with ELISAs. Among the three proteins in common across the two panels (circled), two have higher AUCs and one has a lower AUC in the electrochemiluminescence assays. Based on these observations, higher univariate AUCs likely drive much of the improved performance in the new panel. This effect may result from the increased dynamic ranges and higher sensitivities of the electrochemiluminescense assays. In addition, the use of a much larger dataset in the current study (4435 compared to 922 in the ELISA study) decreased the impact of chance in patient selection, giving us a more robust study and a model that better captures CRC signal in the ITT population.

**Table 6:** Comparison of univariate CRC AUCs for the three proteins appearing in both the ELISA panel [14] and in the new electrochemiluminescense panel

Protein	AUC, ELISA measures	AUC, electrochemiluminescence assay measures
CEA	0.702	0.725
CO9	0.706	0.742
MIF	0.558	0.493

AUCs were calculated for the CRC versus non-CRC discrimination across each study’s full Discovery set (Indeterminate samples were not removed)

### Does the classifier structure reveal anything about cancer biology?

While the primary aim of this study was the development and validation of a clinically useful blood-based CRC test for symptomatic patients, it’s interesting to consider whether the final panel composition or the nature of the final classifier algorithm reveal anything about cancer biology. Our view is that the panel and classifier are suggestive, but that evaluation of these suggestions is outside this study’s scope.

Of the eight protein biomarkers in the new panel, CEA and TFRC are recognized as being involved in gastrointestinal cancers [33]. The remaining six proteins—A1AG, C09, DPPIV, MIF, PKM and SAA—are known to have multiple biological roles including involvement in immune system pathways and as acute-phase proteins whose plasma concentrations increase (positive acute-phase proteins) or decrease (negative acute-phase proteins) in response to inflammation [34–38]. Beyond recognizing these known biological functions, the studies described here do not provide sufficient evidence to hypothesize a mechanism of interaction or connection between these eight proteins identified as CRC predictors.

Our classifier discovery process examined panels and classifiers built using a wide range of feature selection and classifier algorithms, employing a grid approach. We chose this grid approach in part because of the diversity and complexity of cancer biology. The final classifier was a logistic regression, using features selected via penalized regression (GLMNet). What, if anything, does this suggest about the detectability of cancer using blood-based tests?

**Fig. 3:**
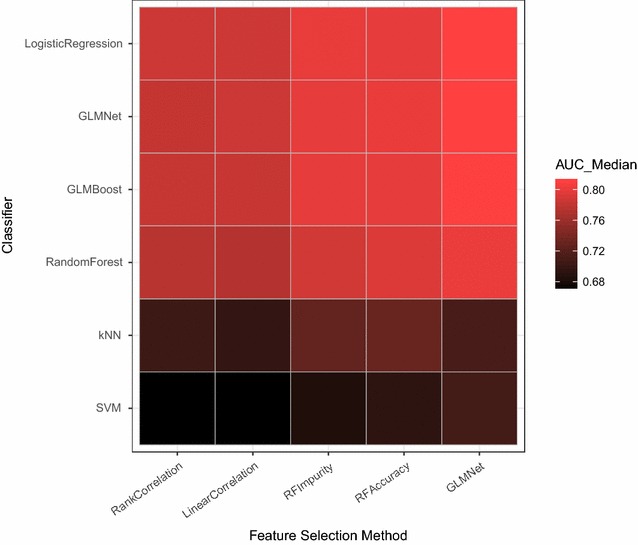
Heatmap illustrating the relative CRC signal found using different combinations of feature selection methods (x-axis) and classifier algorithms (y-axis) across 56,841 classifier builds using ten predictors. The *gray scale* represents the median of Discovery set AUCs (each calculated as the median of the Discovery hold-out test sets, see “Methods” section) found across 147–10,848 builds (with varying algorithm parameters)

Figure 3 presents a heatmap illustrating the AUCs obtained for a range of feature selection and all classifier algorithms used in the study. The linear classifiers—Logistic Regression and GLMNet—combined with the linear feature selection approach (GLMNet) gave the highest performances. These algorithms operate on linear combinations of weighted predictor values, with the outcome class related in a straightforward way to the resulting value (CRC if above some value, otherwise non-CRC). The success of these linear approaches over those that may model more complex mechanisms (e.g. Random Forest) indicates that, regardless of complexity at the cellular level and possible diversity across patients, the pooling of proteins in the bloodstream offers a simplified opportunity for CRC detection. This observation suggests two possibilities: (1) CRC detectability in the blood is dominated by one or more linear mechanisms that mask weaker and possibly more complex and diverse mechanisms, and/or (2) CRC detectability in the blood may be driven only partially by cancer biology per se, and partially by the body’s response to having cancer—for example, an inflammatory or other immune system response. As noted above, evaluation of such suggestions is outside the scope of this study.

## Conclusions

The work presented here builds upon growing interest in understanding the patient population directed to colonoscopies. By focusing on CRC risk stratification within symptomatic patients, the expectation is that the colonoscopy compliance of patients with the apparent highest need can be increased. Other groups [11, 13] have also approached the development of tests for symptomatic patients, though none have yet validated their tests and the tests’ proposed uses have varied. Our view is that CRC tests for symptomatic patients are most helpful when directed to patients who resist colonoscopies despite the presence of symptoms.

The new CRC test presented here also builds upon our earlier work focused on detecting CRC in the symptomatic population. Our initial selection of candidate CRC biomarkers was based on a targeted-mass spectrometry study of samples from asymptomatic patients [17]; that work demonstrated detection of CRC signal in plasma, and uncovered a list of candidate CRC proteins. In subsequently developing a clinical test, we decided to transition from a mass spectrometry platform to an immunoassay platform because of the faster processing time (hours vs days), faster test development time, and the clinical laboratory personnel’s familiarity with the techniques; these considerations made immunoassays a more commercially viable option. This decision was consistent with others’ choices for clinical assay development following mass spectrometry-based discovery programs [39, 40]. We also chose to direct our new test to the CRC-symptomatic population. Given these two choices—a change in assay format and the development of a test for the CRC-symptomatic population—a fresh study was required to identify the top biomarkers for final clinical development. Next, in our first study focused on the symptomatic population, we measured 28 of the CRC candidate proteins with ELISA assays using a case/control design balanced for age and gender [10], yielding an eight protein panel with age- and gender-independent CRC signal in the symptomatic population. We then performed a new study in which we augmented and refined this eight protein ELISA-based panel into a viable clinical test with clinically useful performance in the ITT population [14]. In the current study, we returned to the original list of candidate CRC proteins from our targeted-mass spectrometry studies but chose the multiplexed electrochemiluminescence immunoassay platform; this new platform provided a significantly shorter testing time when compared to standard targeted mass spectrometry methods, and increased sensitivity and dynamic range when compared to ELISAs. Additionally, we brought a different subset of the proteins into the study. We also increased the data set for classifier builds by almost five-fold, from 922 to 4435 samples. In the current study we discovered and validated a new CRC classifier with significantly improved performance as compared to prior results from our and others’ efforts.

This new classifier offers the best validated performance of any blood-based test to clarify CRC risk in symptomatic patients. Results from a test based on this model could serve as evidence in assessing symptomatic patients’ CRC risk and in managing next steps in their care. The results would be particularly useful in guiding the choices of symptomatic patients who resist recommended screening procedures; a positive result on the new test would indicate increased certainty about the usefulness of definitive CRC screening.

## Abbreviations

A1AG: Alpha-1 acid glycoprotein
A1AT: Alpha-1 antitrypsin
AACT: Alpha-antichymotrypsin
AMY2B: alpha-Amylase 2b
ANXA1: Annexin A1
APOA1: Apolipoprotein A-I
CAH1: Carbonic anhydrase 1
CALP: Protein S100-A8/-A9
CATD: Cathepsin D
CEA: Carcinoembryonic antigen-related cell adhesion molecule 5
CLUS: Clusterin
CO3: Complement 3
CO9: Complement 9
CRP: C-reactive protein
DPPIV: Dipeptidyl peptidase IV
ECH1: Delta(3,5)-delta(2,4)-dienoyl-CoA isomerase mitochondrial
FRIL: Ferritin light chain
GARS: Glycine-tRNA ligase
HPT: Haptoglobin
GDF15: Growth differentiation factor 15
GELS: Gelsolin
MIF: Macrophage migration inhibitory factor
OSTP: Osteopontin
PKM2: Pyruvate kinase isozyme M2
PRDX1: Peroxiredoxin-1
PSGL: P-selectin glycoprotein ligand 1
SAA: Serum amyloid A
SBP1: Selenium-binding protein 1
SEPR: Prolyl endopeptidase FAP
SPON2: Spondin-2
TFF3: Trefoil factor 3
TFRC: Transferrin receptor protein
TIMP1: Tissue metalloproteinase inhibitor 1
AUC: area under curve
CRC: colorectal cancer
CV: coefficient of variation
ITT: intent-to-test
LLoQ: lower limit of quantitation
NPV: negative predictive value
PPV: positive predictive value
PQC: process quality control
ROC: receiver operator characteristic
ULoQ: upper limit of quantitation
